# Distribution and Abundance of the World's Smallest Primate, *Microcebus berthae*, in Central Western Madagascar

**DOI:** 10.1007/s10764-014-9768-2

**Published:** 2014-03-28

**Authors:** Livia Schäffler, Peter M. Kappeler

**Affiliations:** 1 Behavioral Ecology & Sociobiology Unit, German Primate Center, 37077 Göttingen, Germany; 2 Present Address: Institute of Regional Development Planning, University of Stuttgart (IREUS), 70569 Stuttgart, Germany

**Keywords:** Biodiversity hotspot, Inventory and monitoring, Lemur conservation, *Microcebus berthae*, Population ecology, Strepsirrhines

## Abstract

The distribution of most recently discovered or described lemur species remains poorly known, but many appear to have small geographical ranges, making them vulnerable to extinction. Research can contribute to future conservation actions on behalf of these species by providing accurate information on local distribution and abundance. The distribution of the world’s smallest primate, the endangered Madame Berthe’s mouse lemur (*Microcebus berthae*), is limited to the Menabe Central region of western Madagascar. This species was discovered in the 1990s, but many fundamental aspects of its ecology remain unknown. The aims of our study were therefore to determine the actual distribution of *Microcebus berthae* across the forests of this region, to estimate population density, and to examine the species’ response to anthropogenic activities. We established 35 1-km line transects across Menabe Central, on which we surveyed mouse lemurs by distance sampling and live trapping. *Microcebus berthae* does not occur in all remaining forests of this small region and its population density is highly heterogeneous, both across its geographic range and locally. Within its area of occupancy, the population of *Microcebus berthae* not only was distributed according to spatial heterogeneities of the habitat, but also responded to anthropogenic disturbances and varied seasonally. Our results indicate that *Microcebus berthae* is susceptible to habitat degradation and avoids human environments spatially. As none of the forest remnants in which the species still occurs were officially protected until recently, immediate conservation actions should focus on effectively protecting Kirindy and Ambadira forests.

## Introduction

Madagascar is a global conservation priority owing to the island’s exceptionally high rates of endemism and considerable anthropogenic threat (Mittermeier *et al.* 1992; Myers *et al.* 2000). Slash-and-burn agriculture to create areas for subsistence agriculture or cattle (zebu) pasture and associated habitat fragmentation represent the major processes affecting Madagascar’s natural forests (Green and Sussman 1990; Irwin and Raharison 2009). These activities have contributed to a reduction of species diversity across all taxa, with characteristic species turnovers from specialists to generalists and from endemics to non-endemics (Irwin *et al.* 2010).

Lemurs are considered the most threatened mammals in the world (Schwitzer *et al.* 2013). Lemur species numbers have multiplied over the past two decades, largely as a result of taxonomic revisions based on genetic studies (Markolf *et al.* 2011). Mouse lemurs of the genus *Microcebus* account for much of that increase, with 21 species presently recognized, most of which inhabit very restricted biogeographic ranges and are therefore highly vulnerable to extinction (Andriantompohavana *et al.* 2006; Kappeler *et al.* 2005; Louis *et al.* 2006, 2008; Olivieri *et al.* 2007; Radespiel *et al.* 2008, 2012; Rasoloarison *et al.* 2000, 2013; Yoder *et al.* 2000). The distribution and conservation status of a majority of newly discovered species remain poorly known, however, as there is a lack of studies assessing lemur ecology and population characteristics across disturbance gradients (Irwin *et al.* 2010).

The western dry deciduous forests of Madagascar are particularly distinctive in terms of high numbers of relict taxa, level of endemism, and intense anthropogenic pressure. These forests are considered one of the world’s most biologically valuable Global 200 ecoregions (Olson and Dinerstein 1998) and one of the most threatened ecosystems globally (Ganzhorn *et al.* 2001).

**Fig. 1:**
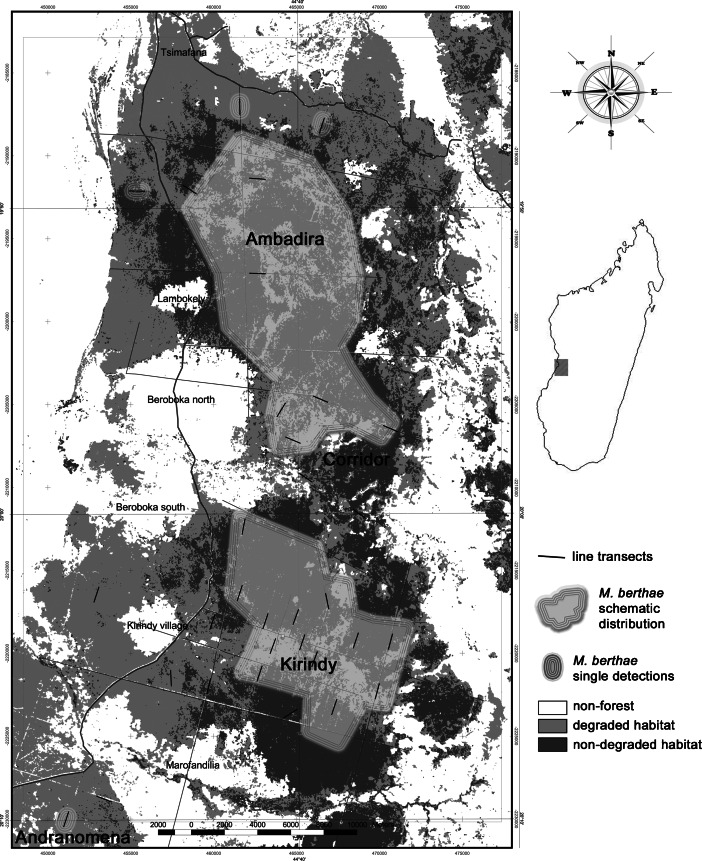
Representation of the regional distribution of *Microcebus berthae* across Menabe Central based on distance sampling and live trapping on line transects during dry and rainy season surveys in Menabe Central from 2003 to 2007.

The largest remnant of Malagasy dry deciduous forest is located in the region of Menabe Central (Fig. 1), which is bound to the north and south by the rivers Tsirihibina and Morondava, respectively (Smith *et al.* 1997; Sorg *et al.* 2003). This area of *ca.* 65,000 ha outranks other Malagasy forests by an exceptionally high rate of local endemism as well as by severe anthropogenic disturbances (Mittermeier *et al.* 1992, Myers *et al.* 2000). The rural human population, comprising several ethnic groups, affects this unique ecosystem via various types of activities (Scales 2012). Forest cover is being reduced by slash-and-burn agriculture, and illegal logging transforms pristine habitat to secondary forest formations, scrub, and savanna (Sorg *et al.* 2003; Zinner and Torkler 2005; Zinner *et al.* 2013). Fragmentation has resulted in three major forest parts of heterogeneous quality.

The two largest remnants, Ambadira and Kirindy Forest, are separated by land cleared over the past decades and are at present connected only by a narrow corridor 5–7 km wide. Although never effectively protected, Ambadira Forest is only moderately accessed by humans and considerable areas of near primary forest persisted (Smith *et al.* 1997). In Kirindy Forest, illegal activities are limited by the presence of a forestry concession of the Centre National de Formation, d’Étude et de Recherche en Environnement et Foresterie (CNFEREF), and the research station of the German Primate Center (DPZ: Kappeler and Fichtel 2012; Sorg *et al.* 2003). The only protected area in Menabe Central, Andranomena Special Reserve, has lost more than two-thirds of the original forest cover since the park’s creation in 1958 and is now largely isolated from the other forest parts (Randrianandianina *et al.* 2003). The Ambadira–Kirindy corridor and Andranomena SR are particularly prone to disturbances owing to their close proximity to villages. Roads cut into the forest for former oil explorations and timber harvesting facilitate public access and exploitation, such as hunting for birds or mammals and the collection of forest products, threatening the survival of endemic species (Réau 2002; Smith *et al.* 1997; Sorg *et al.* 2003). Menabe Central has therefore top conservation priority within one of the “hottest biodiversity hotspots” in the world (Myers *et al.* 2000).

One of the locally endemic species is Madame Berthe’s mouse lemur (*Microcebus berthae*). This species was discovered in 1992 (Rasoloarison *et al.* 2000; Schmid and Kappeler 1994) and represents the smallest primate of the world with an average adult mass of only 33 g. The range of *Microcebus* *berthae* is among the most restricted of all mouse lemur species (Rasoloarison *et al.* 2000; Schwab and Ganzhorn 2004). Within its range, it occupies only habitat patches exceeding 30,000 ha (Ganzhorn *et al.* 2003). *Microcebus berthae* is an ecological specialist with a narrow feeding niche (Dammhahn and Kappeler 2010), and all mouse lemur species face intense predation pressure by nocturnal and diurnal raptors, snakes, and carnivorous mammals (Dammhahn and Kappeler 2008; Goodman 2003; Goodman *et al.* 1993; Rahlfs and Fichtel 2010; Scheumann *et al.* 2007). As a locally endemic specialist with a small biogeographic range that is subject to intense anthropogenic pressure, *Microcebus berthae* meets the conditions for critical conservation (Myers *et al.* 2000) and qualifies as a charismatic flagship species. However, many fundamental ecological aspects remain unknown; in particular, detailed information on the population size and structure is lacking. In this study, we determined the spatial distribution and abundance of *Microcebus berthae* across its entire range and assessed the impact of anthropogenic disturbances on these population parameters.

## Materials and Methods

### Ethical Note

All research reported in this article is in compliance with animal care regulations and applicable national laws of Germany and Madagascar. All research protocols were approved by the appropriate Animal Use and Care committees of Germany (Bundesamt für Naturschutz, BfN) and Madagascar (Ministère de l’Environment et des Eaux et Forêts, MINEEF).

### Lemur Surveys

We established 35 1-km line transects, which were evenly distributed to the extent deemed feasible in the abovementioned forest parts: in total, we sampled 19 transects in Kirindy, 6 in Ambadira, 6 in the corridor connecting the two regions, and 4 in Andranomena SR. As sampling transversally is not possible in dense dry deciduous forest, we used former logging trails or abandoned oil exploration tracks as transects, which ranged between 1 and 3 m in width. We conducted 4 dry season surveys along 34 of these transects (June–September in 2003, 2004, 2006, 2007), and 2 rainy season surveys along 25 transects (February–April and October–December 2007). During each survey, we appraised the population of *Microcebus berthae* on 13–23 transects by distance sampling and sampled the majority of transects twice per survey and several times over subsequent surveys, amounting to a total of 150 1-km samples. All transect walks were conducted between 18:00 h and 20:30 h to control for circadian variation in lemur activity. Two observers slowly walked along each transect, attentively searching for lemurs, with a walking pace standardized to *ca.* 1 km/h, but locally adjusted to habitat and sighting conditions. Detected individuals were identified to the species level and each observer estimated the perpendicular distance from the transect line independently to warrant reliability (Buckland *et al.* 2001).

In addition, we captured lemurs by systematic live trapping along transects to reconfirm the species’ presence/absence pattern. Trapping involved baiting 41 small Sherman live traps (type Sherman LFA: 3 × 3.5 × 9″) set every 25 m and 21 larger live traps every 50 m (type Sherman XLF15: 4 × 4.5 × 15″). All 62 traps were set simultaneously on each transect for three consecutive nights. We started trapping in the dry season 2004, and subsequently trapped on 11–15 transects during each survey, resulting in 63 line-trapping bouts. All captured individuals were briefly restrained and immobilized with Ketanest 100, identified to species level, and a set of standard field measurements (Rasoloarison *et al.* 2000) and tissue samples in form of small ear biopsies were taken for analyses to be reported elsewhere. All subjects were released at the site of capture shortly before dusk at the same day. As we did not obtain the required permit for Andranomena SR, we conducted live trapping only in Ambadira, the corridor, and Kirindy.

### Habitat Classification

To take differential habitat suitability and disturbance levels into account, we assessed the degradation of the forest surrounding each transect based on stand and understory density, canopy height and cover, as well as decades of combined familiarity with this region and its forests. Our ground-based appraisal of forest quality allowed for reliable classification only into non-degraded and degraded habitat. The two categories broadly matched with a forest classification based on a Landsat ETM 7 satellite image (Zinner and Torkler 2005; Fig. 1) and we therefore consider them reliable.

### Data Analyses

We estimated population densities in DISTANCE 6.0 (Thomas *et al.* 2009) based on distance sampling data. Assuming that the detection probability on the transect line equals 1, DISTANCE fits a detection function to the histogram of observed distances and compensates for missed detections as well as for failed identification at greater distance from the transect. To validate distance data, we visually inspected detection curves of identified and non-identified objects, i.e., the distribution of detection events plotted against perpendicular distance from the transect line, and corrected for shortcomings by appropriate data handling. One basic assumption of distance sampling is the distribution of transects across the entire habitat, rather than exclusively along trails, as edge effects may corrupt density estimation (Buckland *et al.* 2001). Small sample sizes prevented a direct test of varying transect widths on the encounter rates of *Microcebus berthae*, but we could rule out an effect in closely related gray mouse lemurs (*Microcebus murinus*; Schäffler 2012) and transect type did not have any influence on encounter rates in other cheirogaleids (Johnson and Overdorff 1999; Lehman 2006).

Encounter rates did not differ significantly between repeated transect walks within single surveys in any of the study regions, or between replicate surveys within the same season; neither did we find differences in encounter rates between years, or between seasons in Kirindy Forest (Schäffler 2012). Moreover, encounter rates during the late rainy season did not differ from those of the early rainy season (Wilcoxon signed-ranks test: *Z* = –0.282, *N* = 16, *P* = 0.778). We therefore pooled encounter rates over repeated transects walks and over consecutive surveys.

### Density Estimation in DISTANCE 6.0

The detection function depends on a species’ visual conspicuousness as well as on sighting conditions, which can differ considerably in deciduous habitats. To account for seasonal differences in detection probabilities and potential temporal heterogeneities in lemur distribution, we calculated the detection function for dry and rainy season separately by including “season” as a covariate in the multiple covariate distance sampling engine (MCDS; Thomas *et al.* 2010). Transects represented “samples” in DISTANCE, surveys were treated as “strata,” and all samples across Menabe Central were embodied in the “global level.”

After data exploration, we appropriately grouped raw distance data and discarded the largest 5% to offset shortcomings, such as accumulation of estimated perpendicular distances at 5 m, 10 m, 15 m, and 20 m, as well as outliers detected at great distances from transect lines (Buckland *et al.* 2001). The size of distinct forest regions were set to 200 km^2^ for Ambadira, 35 km^2^ for the corridor, 200 km^2^ for Kirindy (125 km^2^ in the dry season 2003 survey), and 65 km^2^ for Andranomena SR. As our reliability analyses justified pooling of repeated transect walks, we entered cumulative counts per transect and corrected for sampling effort. Both models provided in MCDS (half-normal and hazard-rate key functions) were tested with all available series expansions. Models were chosen by visual inspection of the detection function’s fit to histograms of distance data and by lowest Akaike’s information criterion (AIC; Buckland *et al.* 2001). Population densities and encounter rates were estimated in DISTANCE by survey unit (stratum level) and for the entire survey (global level). To obtain regional density estimates, we calculated means of survey-wise density estimates given by DISTANCE for each of the forest regions and for dry and rainy season respectively (Thomas *et al.* 2010).

### Determinants of Spatial Distribution and Abundance

We conducted further analyses based on encounter rates, as they are based on fewer assumptions than density estimates. We analyzed the regional distribution of *Microcebus berthae* for impacts of habitat degradation and human frequentation. Dry and rainy season data were analyzed separately to document responses to temporal variations in food supply.

To describe the regional population distribution of *Microcebus berthae* in Menabe Central, we examined the species’ distribution across the four distinct forest parts. We tested specific encounter rates by season for differences between the regions (Kruskal–Wallis ANOVA). In total, we surveyed 34 single transects during the dry season (Ambadira *N* = 5, corridor *N* = 6, Kirindy *N* = 19, Andranomena *N* = 4), and 25 during rainy season surveys (Ambadira *N* = 4, corridor *N* = 6, Kirindy *N* = 11, Andranomena *N* = 4).

To quantify the influence of habitat suitability on population densities, we tested specific encounter rates by season for differences between degraded and non-degraded habitat (Mann–Whitney *U*-test). In addition, we examined the proportions of transects in degraded and non-degraded habitat on which *Microcebus berthae* was present/absent. We tested 16 dry season transects in non-degraded and 18 in degraded habitat, as well as 11 in non-degraded and 14 in degraded forest transects surveyed during the rainy season for differences in the proportions of transects colonized (χ^2^-test).

Under the assumption that human impact is strongest close to villages and declining with increasing distance, we used the distance to the closest village as an inverse proxy for anthropogenic influence. Spearman rank correlations assessed the relationship between *Microcebus berthae* encounter rates and the distance of transects from the next village separately for each season (dry season *N* = 34; rainy season *N* = 25). To exclude redundancy of variables, we tested degraded and non-degraded habitat transects for differences in distance from the nearest village (Mann–Whitney *U*-test).

Density estimates based on capture rates do not reflect true population densities for several reasons. First, food availability determines trapability. Trapping success in lemurs rises with decreasing productivity (Lahann *et al.* 2006), and increased trapability in less productive areas may compensate for low population sizes. In response to a seasonal increase in resource abundance, mouse lemurs enter traps less frequently during the rainy season (Ganzhorn and Schmid 1998). Second, habituation to traps may cause bias. As our trap lines ranged from frequently baited to never sampled before, capture data were not suited for population density estimates and were consequently employed only to reaffirm the observed presence/absence pattern.

## Results

### Survey Results

We detected 109 *Microcebus berthae* individuals and recorded a gradient in regional abundance across forest regions: We encountered most individuals in Ambadira, considerably fewer in the corridor and in Kirindy Forest, and the species was virtually absent from Andranomena Special Reserve (with only a single detection). However, regional differences in *Microcebus berthae* encounter rates were not statistically significant during either the dry (Kruskal–Wallis test: *H*(3, *N* = 34) = 4.080, *P* = 0.253) or the rainy season (*H*(3, *N* = 25) = 4.246, *P* = 0.236).

*Microcebus berthae* was present only on a fraction of our line transects. During the dry seasons, we encountered *Microcebus berthae* on 50% of surveyed transects (*N* = 34) and on only 40% during rainy seasons (*N* = 25). The area of occupancy was considerably smaller than previously assumed based on geographic range borders (Fig. 1), and the extent of occurrence therefore does not allow us to infer the species’ global population size.

### Distance Samples

**Fig. 2:**
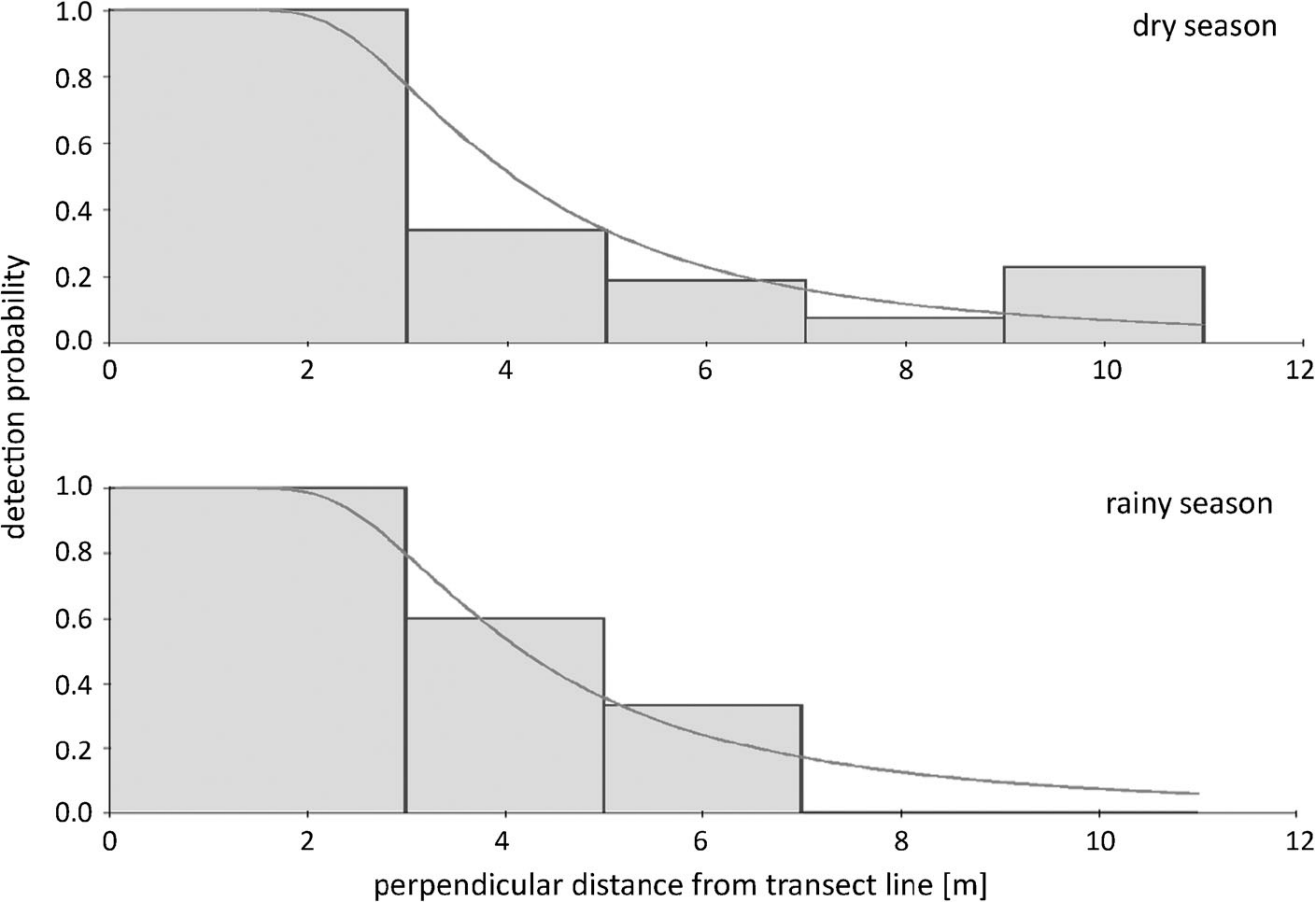
Distance data for *Microcebus berthae* after removal of outliers (gray bars; *N* = 101) and corresponding detection functions (fitted in DISTANCE 6.0: black lines) during dry and rainy season surveys in Menabe Central from 2003 to 2007.

Appropriate grouping and truncation of outliers fixed shortcomings of data, and detection curves of identified objects subsequently conformed to the shape criterion, one of the major assumptions of distance sampling (Buckland *et al.* 2001; Fig. 2). Detection curves of non-identified *Microcebus* allowed us to compensate for failed identifications in the same way as missed detections in DISTANCE.

### Population Density Estimation

Sample sizes of *Microcebus berthae* exceeded the 60–80 detection events required for density estimation (Thomas *et al.* 2010), and distance data allowed for a good fit of detection functions. Rainy season detection probabilities were generally lower than in the dry season and declined more rapidly with increasing distance from the transect line (Fig. 2). The derived hazard-rate model provides a good fit of the detection function to genuinely spiked data (Buckland *et al.* 2001). Lowest AIC affirmed best fit of the hazard-rate key function to *Microcebus berthae* distance data, whereas none of the series expansions improved model fit.

Hazard-rate key function: $1- \exp \left(-{\left(\frac{x}{\sigma (z)}\right)}^{-b}\right)$

Parameter values for distance data for *Microcebus berthae*:

*x = 11* m: distance from transect line
*b = 2.519*: power parameter
σ *= 3.618*: scale parameter, controlling the width of the detection function; is modeled as an exponential function of the covariates:
  $$ \sigma (z)={\beta}_0\ast \exp \left({\beta}_1{z}_1+{\beta}_2{z}_2+{\beta}_3{z}_3\right) $$
  β_0_: intercept of the scale parameter σ
  *z*: covariate = 0.02974
  β: covariate parameters

Population densities of *Microcebus berthae* across Menabe Central were estimated by DISTANCE to 80.3 individuals/km^2^ (39.23% CV). Multiplying by the sampled area of 500 km^2^ yields a total population size of *ca.* 40,000 individuals. For a rough impression of the species’ regional distribution, see unweighted means of stratum level density estimates per forest regions (pooled within seasons over repeated surveys) in Table I.

**Table I:** Population density estimates for *Microcebus berthae* by forest region and season; unweighted means of survey-wise density estimates per forest part for Ambadira (2004–2007) and Kirindy Forests (2003–2007), and survey-wise density estimates for the corridor sampled once per season in 2007

Forest region	Season	Density of *Microcebus berthae* (individuals/km^2^)	SD	*N* (surveys)
Ambadira	Dry	94.6	60.8	2
	Rainy	180.5	145.9	2
Kirindy	Dry	54.8	27.1	4
	Rainy	57.3	16.2	2
Corridor	Dry	68.8	0.0	1
	Rainy	34.4	0.0	1

### Determinants of the Regional Distribution of *Microcebus berthae*

**Fig. 3:**
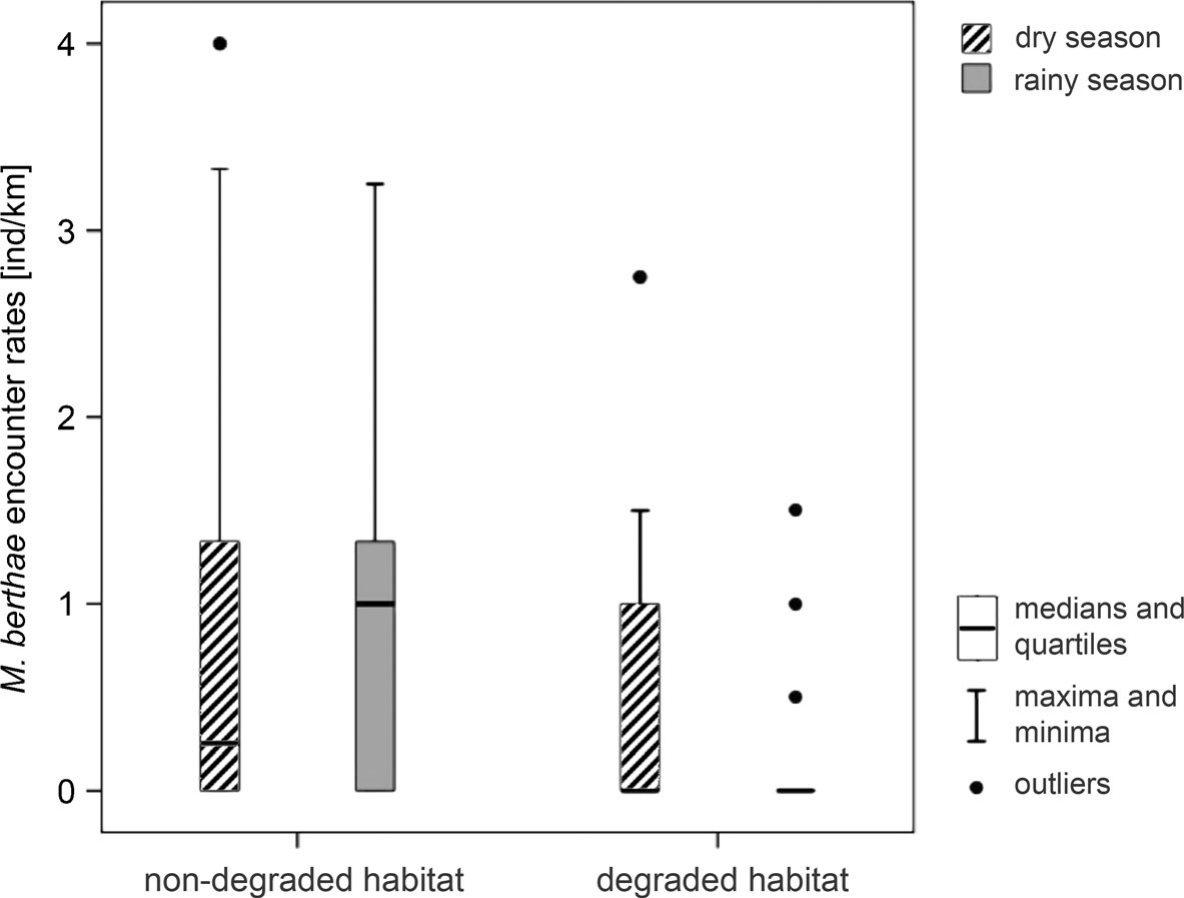
Encounter rates of *Microcebus berthae* in non-degraded and degraded habitat during four dry season (2003, 2004, 2006, 2007; hatched bars; *N* = 34) and two rainy seasons (both in 2007; gray bars; *N* = 25) surveys in Menabe Central.

**Fig. 4:**
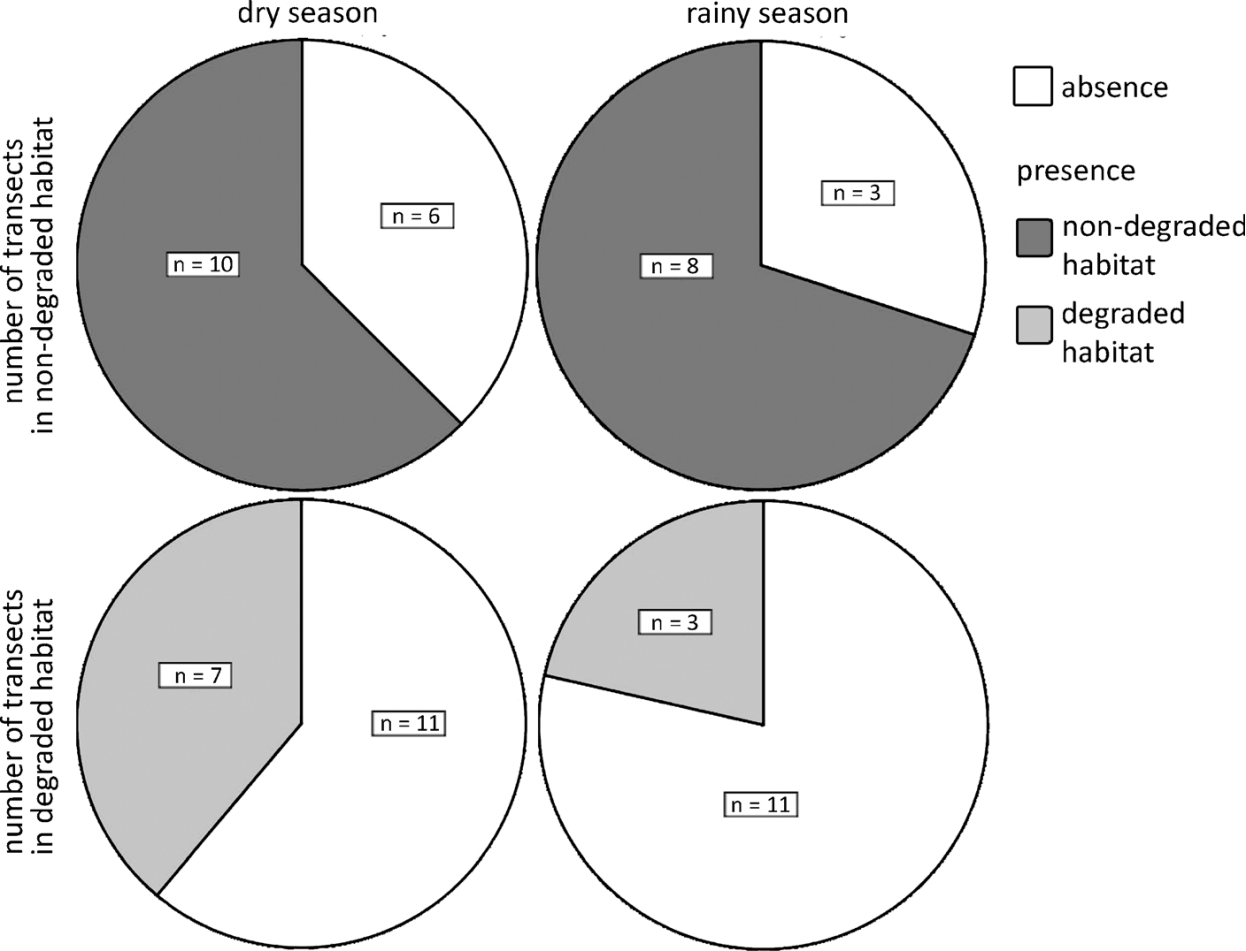
Number of transects in non-degraded (dark gray) and degraded habitat (light gray) on which *Microcebus berthae* was present/absent during four dry (2003, 2004, 2006, 2007; *N* = 34) and two rainy seasons (both in 2007; *N* = 25) surveys in Menabe Central.

In the rainy season, we found a non-significant trend towards higher encounter rates on non-degraded than on degraded habitat transects (Mann*–*Whitney *U*-test: MWU_11,14_ = *–*2.199, *P* = 0.051). Dry season encounter rates did not differ between non-degraded and degraded habitat (MWU_16,18_ = *–*0.997, *P* = 0.365; Fig. 3). In either season, *Microcebus berthae* was present on the majority of transects in non-degraded forest, but only on a small number of transects in degraded habitat (Fig. 4). In line with encounter rates, the proportions of transects occupied by *Microcebus berthae* in non-degraded and degraded habitat differed only during the rainy (chi-squared test: χ^2^ = 4.573, df = 1, *P* = 0.032), but not during the dry season (χ^2^ = 1.889, df = 1, *P* = 0.169). Line trapping reconfirmed the observed distribution pattern and supported the species’ essential confinement to non-degraded habitat. With respect to anthropogenic environments, we encountered significantly more *Microcebus berthae* with increasing distance from villages during the dry season (Spearman’s rank correlation: *r* = 0.529, *N* = 34, *P* = 0.001), but not during the rainy season (*r* = 0.268, *N* = 25, *P* = 0.098). Non-degraded and degraded habitat transects did not differ significantly in distance from the nearest village (MWU_16,19_ = *–*1.457, *P* = 0.145).

## Discussion

### Spatial Distribution and Abundance

The regional distribution of *Microcebus berthae* across forest regions and across transects reflected high ecological specialization and confirmed this species’ sensitivity to anthropogenic disturbances, as it was virtually absent from Andranomena SR and west of the Route National 8. Anthropogenic impact on the forest has been most severe in the southwestern part of Menabe Central (Smith *et al.* 1997), where the Route National traverses the forest and inhabitants of several villages harvest or clear the forest for subsistence (Réau 2002). Low forest accessibility combined with relatively low human population densities may explain why Ambadira Forest is the major stronghold of *Microcebus berthae* despite a lack of effective protection. Our overall density estimate of 80 individuals/km^2^ for Menabe Central is likely to be reliable, but estimates for particular forest parts should be regarded with caution as they represent unweighted means of survey-specific results.

As in other vertebrate specialists with narrow ecological niches (Swihart *et al.* 2003, 2006), population densities of *Microcebus berthae* were highest in most suitable core areas in greater distance from its range boundary. Both distance sampling and line trapping revealed that only a fraction of transects were colonized and the population was confined largely to non-degraded habitat. During the rainy season, a majority of the population concentrated in non-degraded habitat, where increases in carrying capacity are presumably most pronounced. In addition, intact habitat may provide structural characteristics that are of particular significance for breeding in the rainy season (liana and a high herb layer cover required for nest building: Rendigs *et al.* 2003). During the dry season, the population appears to spread out to degraded habitat. This is in accordance with the compensation of resource scarcity by feeding on secretions of homopteran larvae (Dammhahn and Kappeler 2008, 2010), which aggregate along forest edges (Corbin and Schmid 1995). The vicinity of villages negatively affected the distribution of *Microcebus berthae* predominately during the dry season, when increased forest accessibility is assumed to favor human frequentation (Smith *et al.* 1997).

We can rule out two alternative explanations for the divergent results from the rainy and the dry season: First, rainy season foliage should hamper visibility more drastically in non-degraded than in degraded habitat. However, we encountered more *Microcebus berthae* and the species was present on a higher number of transects in non-degraded habitat during the rainy season. The vicinity of villages should not have any effect on detectability as degraded and non-degraded habitat transects did not significantly differ in distance from the nearest village. Second, an increase in population density after the breeding season should not be limited to non-degraded habitat. Moreover, we conducted our two rainy season surveys before and after the birth season, respectively, and did not find differences between pre- and post-birth season encounter rates. The observed seasonal differences are therefore most likely explained by local movements of individuals, which are a widespread response of mammalian dietary specialists that need to cope with seasonally dry tropical forests (Stoner and Timm 2011). In conclusion, *Microcebus berthae* appears not only to respond to pronounced spatial heterogeneities, but also to track temporal heterogeneities in resource supply as well as in anthropogenic disturbances across its area of occupancy.

### Threats to the Persistence of *Microcebus berthae*

Sensitivity to fragmentation is evident in *Microcebus berthae* as viable populations are supported only in the core areas of large fragments in Menabe Central, and the species is therefore at major risk of extinction (Gibbons and Harcourt 2009). Lemurs disappear from fragments of decreasing size in predictable sequence (Ganzhorn 1999; Ganzhorn and Eisenbeiß 2001; Ganzhorn *et al.* 1999; Irwin and Raharison 2009; Irwin *et al.* 2010), and, once lost, do not reappear in impoverished communities (Ganzhorn *et al.* 2003).

Given the species’ susceptibility to anthropogenic disturbances, it is imperative to prevent further forest loss, fragmentation, and degradation of remnant habitat to preserve the exceptional lemur diversity of Menabe Central. This urgently requires implementation and effective control of the new protected area status for Menabe Central. Conservation actions should focus on Kirindy and Ambadira forests, as well as on the corridor in order to prevent isolation of individual habitat patches. As species collapses in Madagascar have been documented even in relatively large forest stands, in which fragmentation and forest edges were not yet obvious (Ganzhorn *et al.* 2001), populations should also be continuously monitored to determine viability and to distinguish them from remnant or sink populations (Irwin *et al.* 2010).

In the long run, protection of remnant forest cover will not be sufficient to maintain Menabe Central’s exceptional biodiversity as complete isolation prevents the great majority of species from shifting their ranges in response to global warming (Soulé *et al.* 2004). Habitat connectivity is of prime importance for ecosystem resilience in view of climate change, but dry forest restoration in Madagascar is highly challenging not only because of sociopolitical factors: Secondary dry forests are characterized by vegetation comprising a subset of the original flora that relies predominately on wind dispersal, and habitat content is relatively inhospitable to animals and more deciduous than forests that have succeeded through vertebrate seed dispersal (Janzen 1988). Climate change will further aggravate the inherent aridity (Hannah *et al.* 2008) that hampers natural regeneration of endemic wood taxa (Hunziker 1981, in Réau 2002).

Our extensive line transect survey proved appropriate for large-scale population assessments of small arboreal primates and for thorough analyses of species distribution and abundance. Many of the earlier lemur surveys aimed to generate species inventories (Rakotoarison *et al.* 1993; Thalmann and Rakotoarison 1994) or to confirm the occurrence of species (Rakotonirina *et al.* 2013). Rapid assessments and the use of simple presence/absence or count data prevented density estimation in most cases (Hawkins *et al.* 1998), and only few studies investigated the relationship between lemur distribution and environmental or anthropogenic factors (Hawkins *et al.* 1990; Sterling and Ramaroson 1996). Our presence/absence data from visual detections and live trapping on line transects were useful to determine our focal species’ area of occupancy within the presumed geographic range borders, but only the analysis of transect specific encounter rates revealed the highly heterogeneous distribution of *Microcebus berthae* and allowed us to understand the spatial population structure against the backdrop of anthropogenic disturbances. In contrast to earlier population density estimates, which were mostly based on strip sampling (Irwin *et al.* 2001, 2005; Sterling and Rakotoarison 1998; *cf.* Banks 2002; Kelley *et al.* 2007), our modeling of line transect detections in DISTANCE provides an advanced estimate of mean population density across the species’ entire biogeographic range, and consequently of total population size. Moreover, with >100 detections, our long-term survey prepared the ground for continued population monitoring of *Microcebus berthae*. With a specific mean detection rate of 0.6 individuals/km, >100 km of transect walks were necessary to obtain the minimum number of detections required for fitting a detection function in DISTANCE (Thomas *et al.* 2010). Distance data from subsequent rapid surveys can now be added to our database to estimate survey-wise population densities at reasonable costs. However, unfortunately, there are no shortcuts for similar future studies of other species unless encounter rates are considerably higher than in *Microcebus berthae*.
